# Supplementary material for: High resolution multispectral spatial light modulators based on tunable Fabry-Perot nanocavities
Source: Light Sci Appl. 2022 May 17;11:141. doi: 10.1038/s41377-022-00832-6 (PMC9114107; doi:10.1038/s41377-022-00832-6)
Supplement: Supplementary file 1 — Supplementary Information [file 41377_2022_832_MOESM1_ESM.docx]

**Supplementary Information for**:

High Resolution Multispectral Spatial Light Modulators based on Tunable Fabry-Perot Nanocavities

*Shampy Mansha^⊥^, Parikshit Moitra^⊥^, Xuewu Xu^⊥^, Tobias W. W. Mass^⊥^, Rasna Maruthiyodan Veetil , Xinan Liang, Shi-Qiang Li, Ramón Paniagua-Domínguez* and Arseniy I. Kuznetsov**

*Institute of Materials Research and Engineering, A*STAR (Agency for Science, Technology and Research), 138634, Singapore*

*Corresponding authors:* [*ramon_paniagua@imre.a-star.edu.sg*](mailto:ramon_paniagua@imre.a-star.edu.sg)*;*

[*arseniy_kuznetsov@imre.a-star.edu.sg*](mailto:arseniy_kuznetsov@imre.a-star.edu.sg)

**
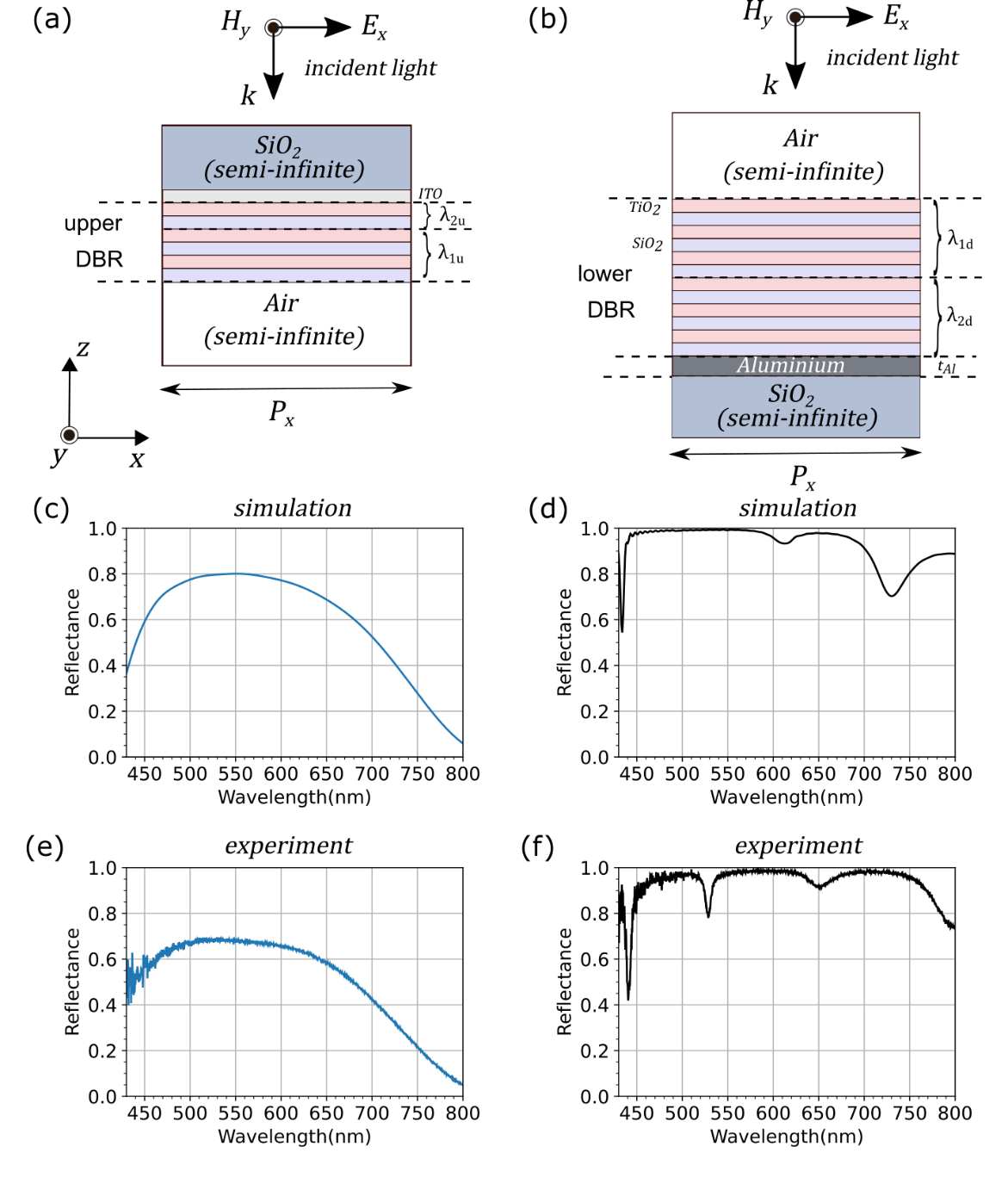
**

**Figure S1.** **Reflectance spectra of upper and lower DBRs**: (a) Schematic of the upper DBR, with a semi-infinite layer of glass on the top and a semi-infinite layer of air at the bottom (ITO layer thickness is $23 nm$). (b) Schematic of the lower DBR, with a semi-infinite layer of air on the top and a semi-infinite layer of glass at the bottom (Al thickness is $t_{Al}=150 nm$). In both (a) & (b) there are alternate layers of TiO2/SiO2 ($\lambda/4$: $\lambda_{2u}=580 nm,$ $\lambda_{1u}=500 nm,$ $\lambda_{1d}=450 nm,$ $\lambda_{2d}=530 nm$) [$\lambda$ denotes the four target wavelengths for calculating thickness of dielectric layers in DBR stack: where $\lambda_{2u}$ and $\lambda_{1u}$ are for upper DBR stack; and $\lambda_{1d}$ and $\lambda_{2d}$are for bottom DBR stack (see Methods: Simulation methodology and optimization of Distributed Bragg Reflector, for more details)] (c), (e) Reflectance spectra of the upper DBR from experiment and simulation, respectively. (d), (f) Reflectance spectra of the lower DBR from experiment and simulation, respectively.


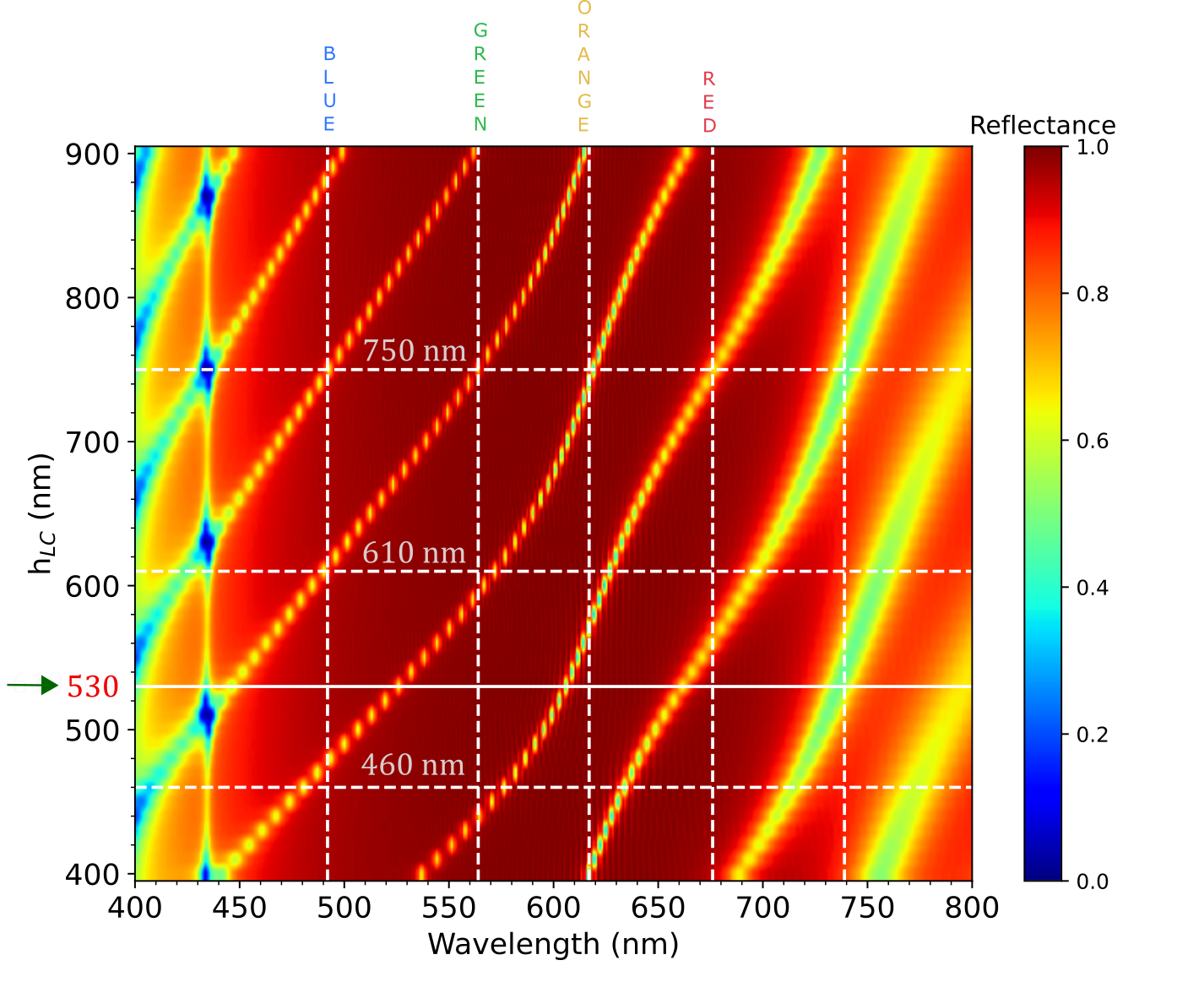


**Figure S2.** **Reflectance colormap of the FP-SLM obtained from simulations for different heights of the cavity (**$\boldsymbol{h}_{\boldsymbol{LC}}$**) and wavelengths** (non-pixelated case $P=1.14 um, gap=0 nm$[where $P$ is the pixel pitch and gap is the inter-pixel gap between adjacent Al electrodes at the bottoms]). Three different heights can be identified $h_{LC}=460 nm, 610 nm, 750 nm$ (dashed white horizontal line) where there is possibility of achieving RGB. The green arrow and the thick white horizontal line indicates $h_{LC}=530 nm$, which is the estimated height of the FP-SLM device in the experiment.


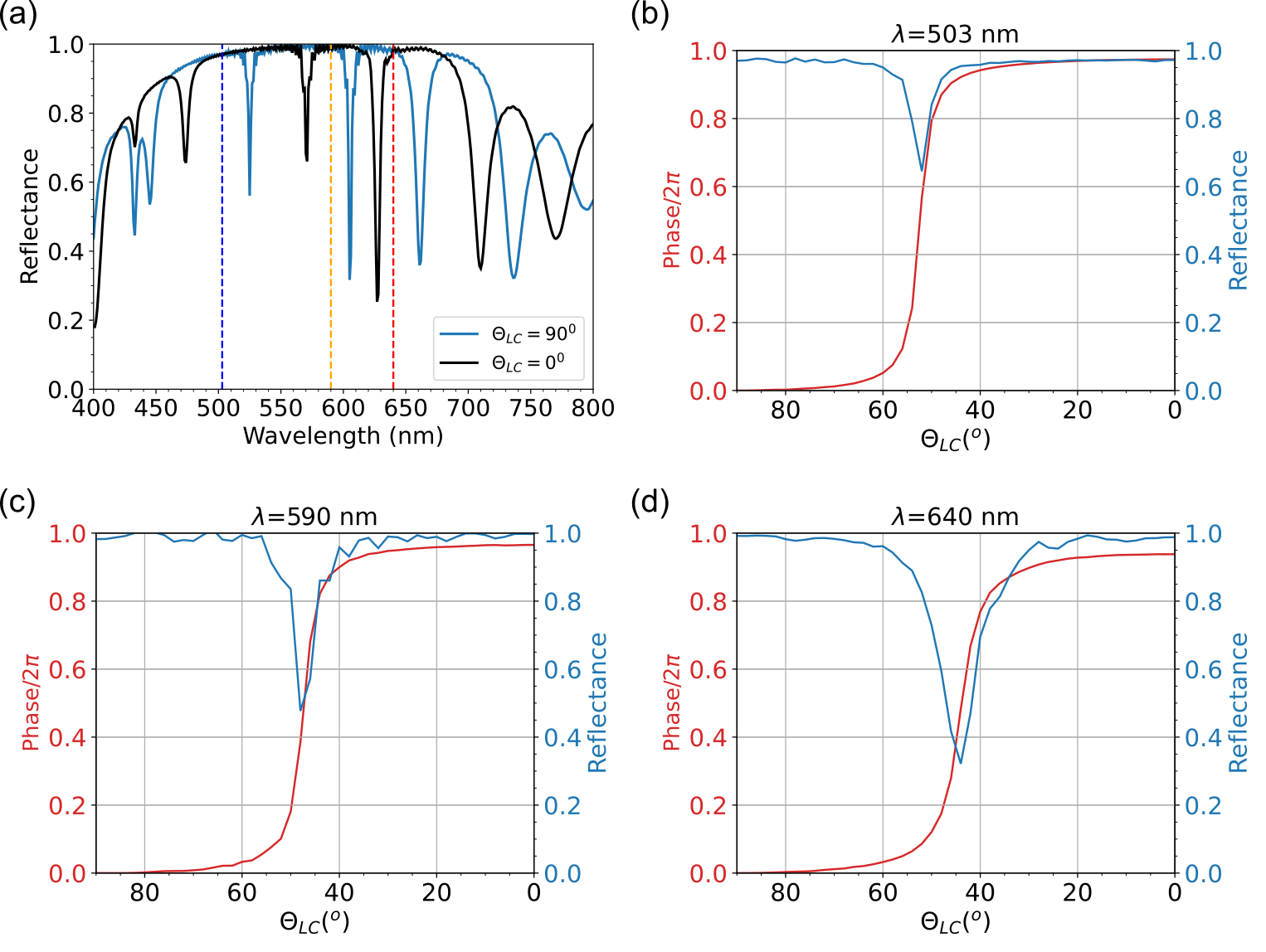


**Figure S3.** **Reflectance & phase shift in simulation,** $\boldsymbol{h}_{\boldsymbol{LC}}\boldsymbol{=530 nm}$. (a) Reflectance spectra for the two cases $\Theta_{LC}={90}^{0}$ & $\Theta_{LC}=0^{0}$. The dashed vertical lines represents the selected wavelengths in blue: 503 nm, orange: 590 nm, red: 640 nm parts of the spectrum. (b)-(d) The reflectance and phase shift plots at the selected wavelengths (as shown in (a)) of 503 nm, 590 nm & 640 nm respectively.

**
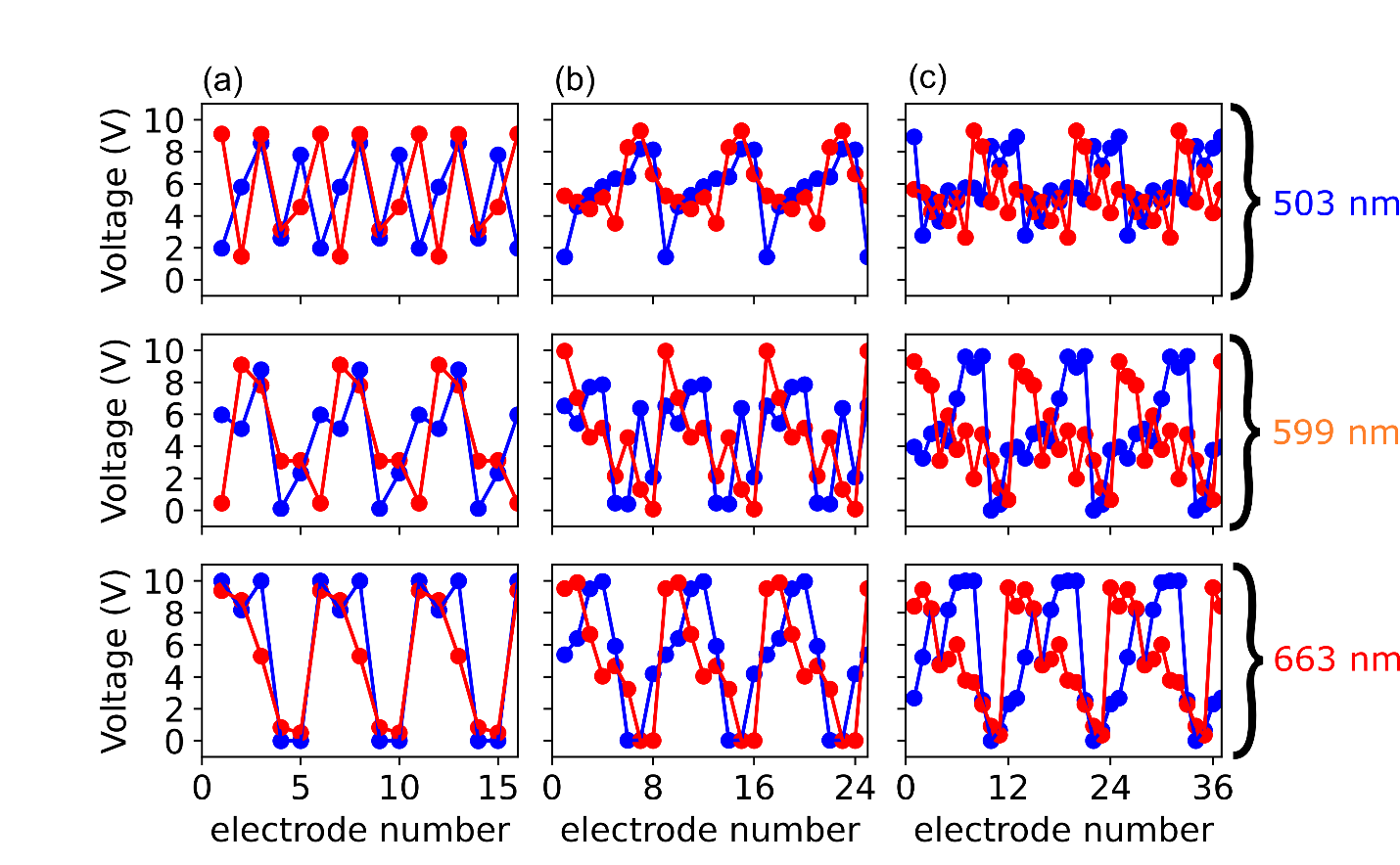
**

**Figure S4.** **Optimized voltage profiles for programmable beam steering.** Rows from top to bottom correspond to different wavelengths, as indicated on the right. Columns (a) – (c) show the optimization results for supercell sizes of 5, 8 and 12 electrodes, respectively. Red voltage values correspond to bending into +1 diffraction order and blue voltage values the reverse bending, i.e. into -1 order. This color code corresponds to Fig. 4 in the main text.


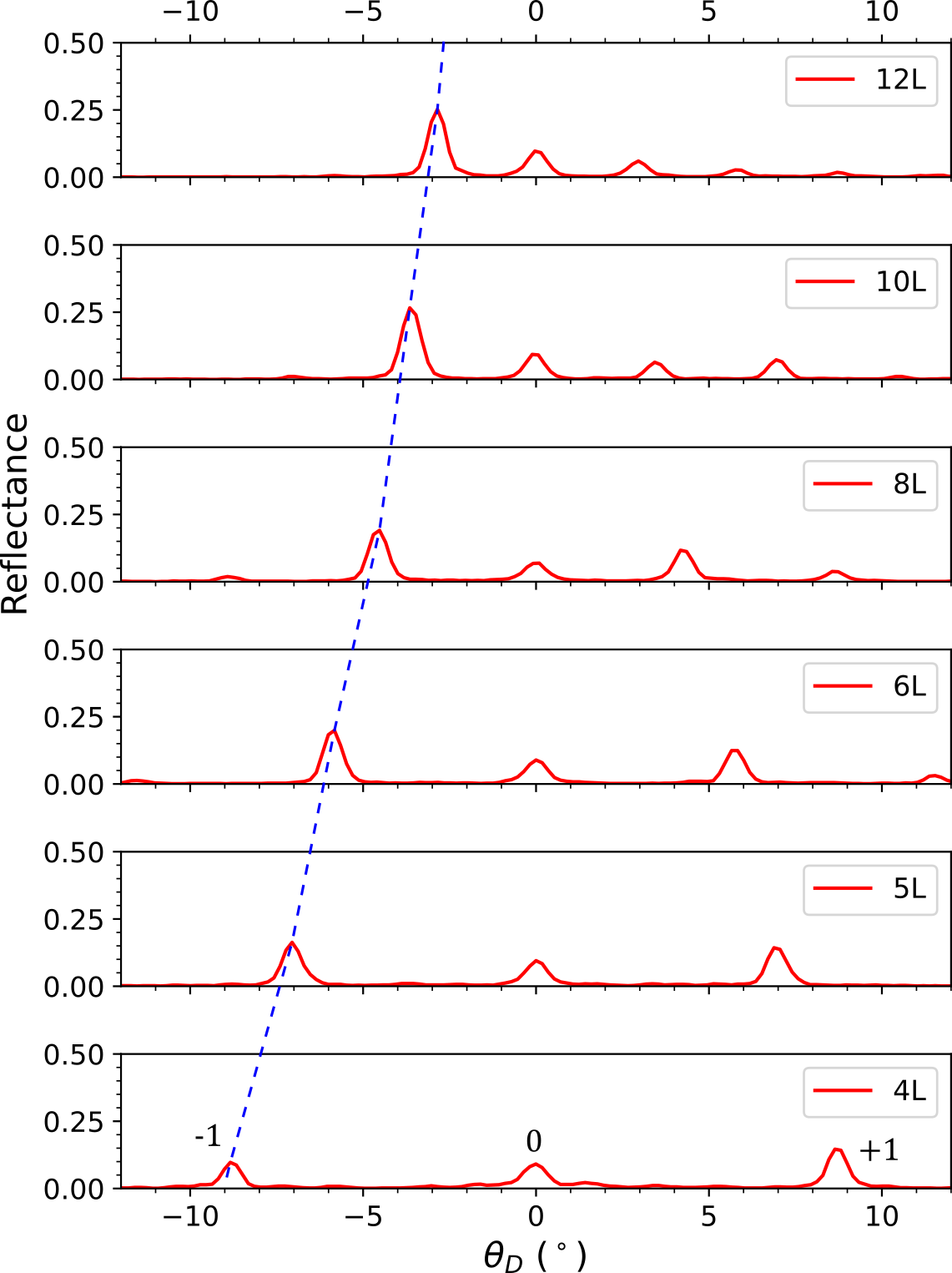


**Figure S5. Programmable beam steering in a wide range of device configurations.** Experimentally measured efficiency as a function of the diffraction angle for the FP-SLM operating at 663 nm. From bottom to top: 4-pixel supercell (4L), 5-pixel supercell (5L), 6-pixel supercell (6L), 8-pixel supercell (8L), 10-pixel supercell (10L) and 12-pixel supercell (12L). The blue dashed line is a guide to the eye tracking the main diffraction order to which power is channeled.


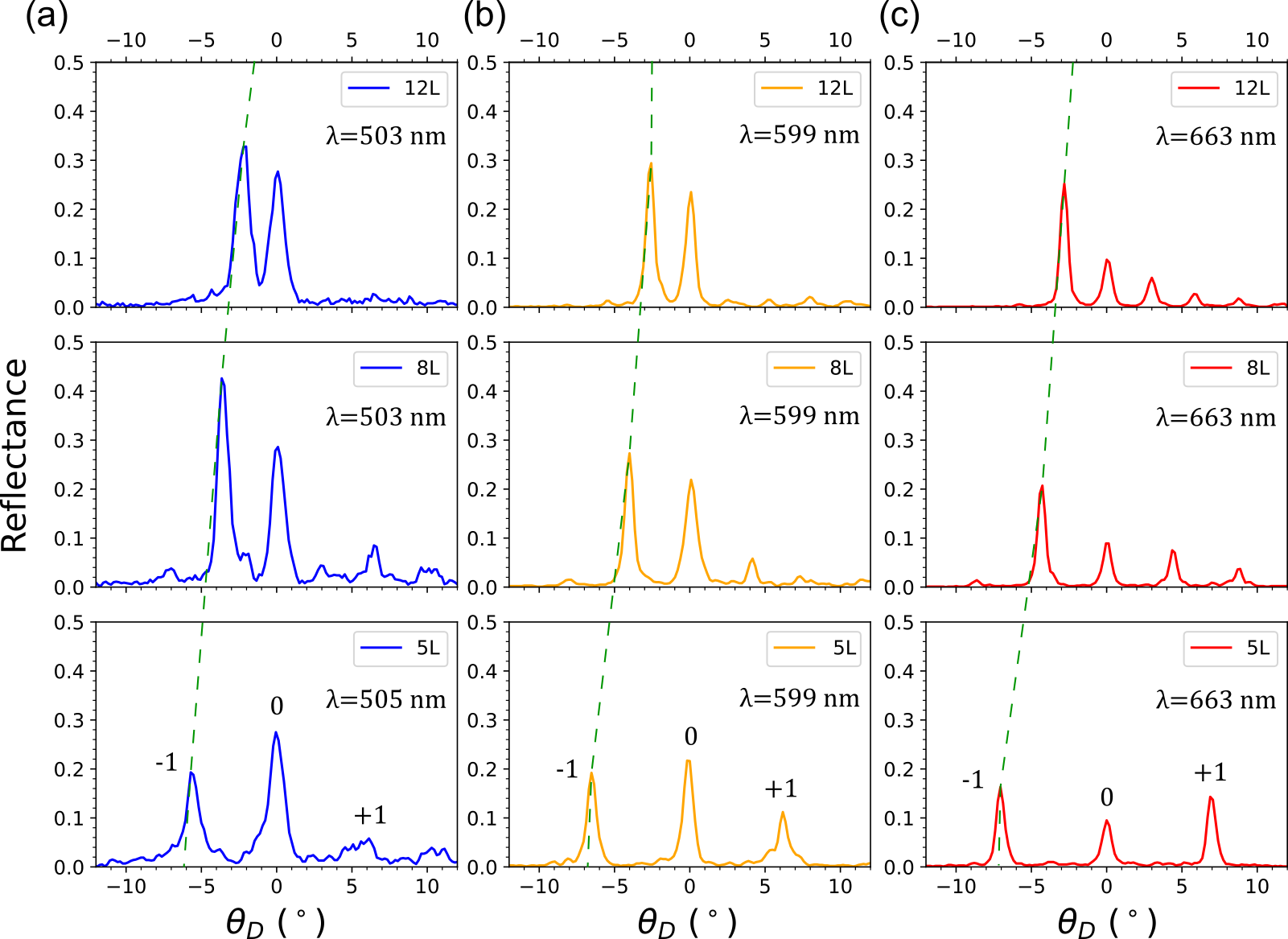


**Figure S6.** **Multi-spectral programmable beam steering.** Experimentally measured efficiency as a function of the diffraction angle for the FP-SLM operating at wavelengths in the blue (a), orange (b) and red (c) spectral regions. From bottom to top: 5-pixel supercell (5L), 8-pixel supercell (8L) and 12-pixel supercell (12L). The green dashed lines are guides to the eye tracking the main diffraction order to which power is channeled.


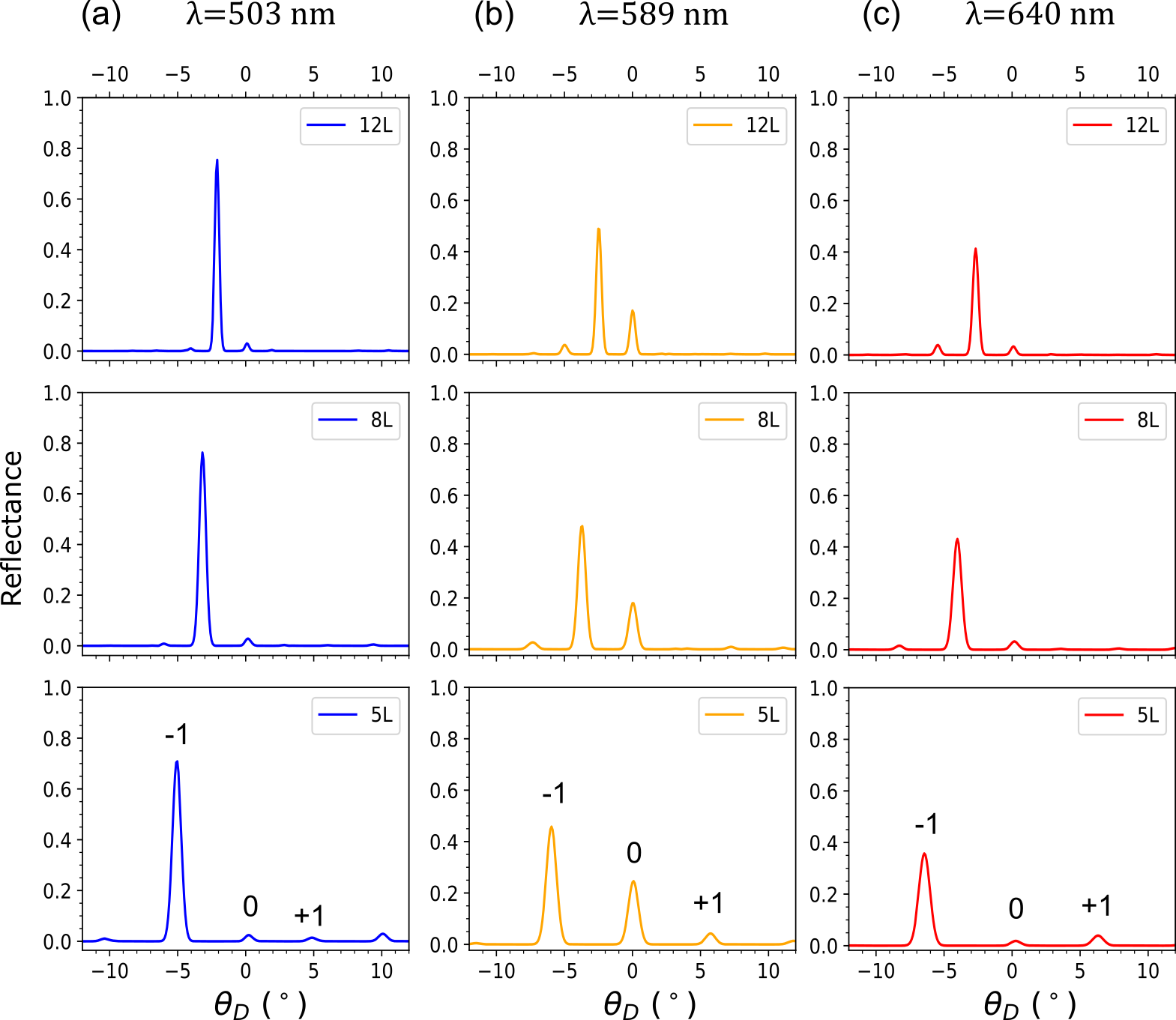


**Figure S7.** **Simulated multi-spectral programmable beam steering.** Simulated reflectance as a function of the diffraction angle for the FP-SLM operating at wavelengths in the blue (a), orange (b) and red (c) regions. From bottom to top: 5-pixel supercell (5L), 8-pixel supercell (8L) and 12-pixel supercell (12L).


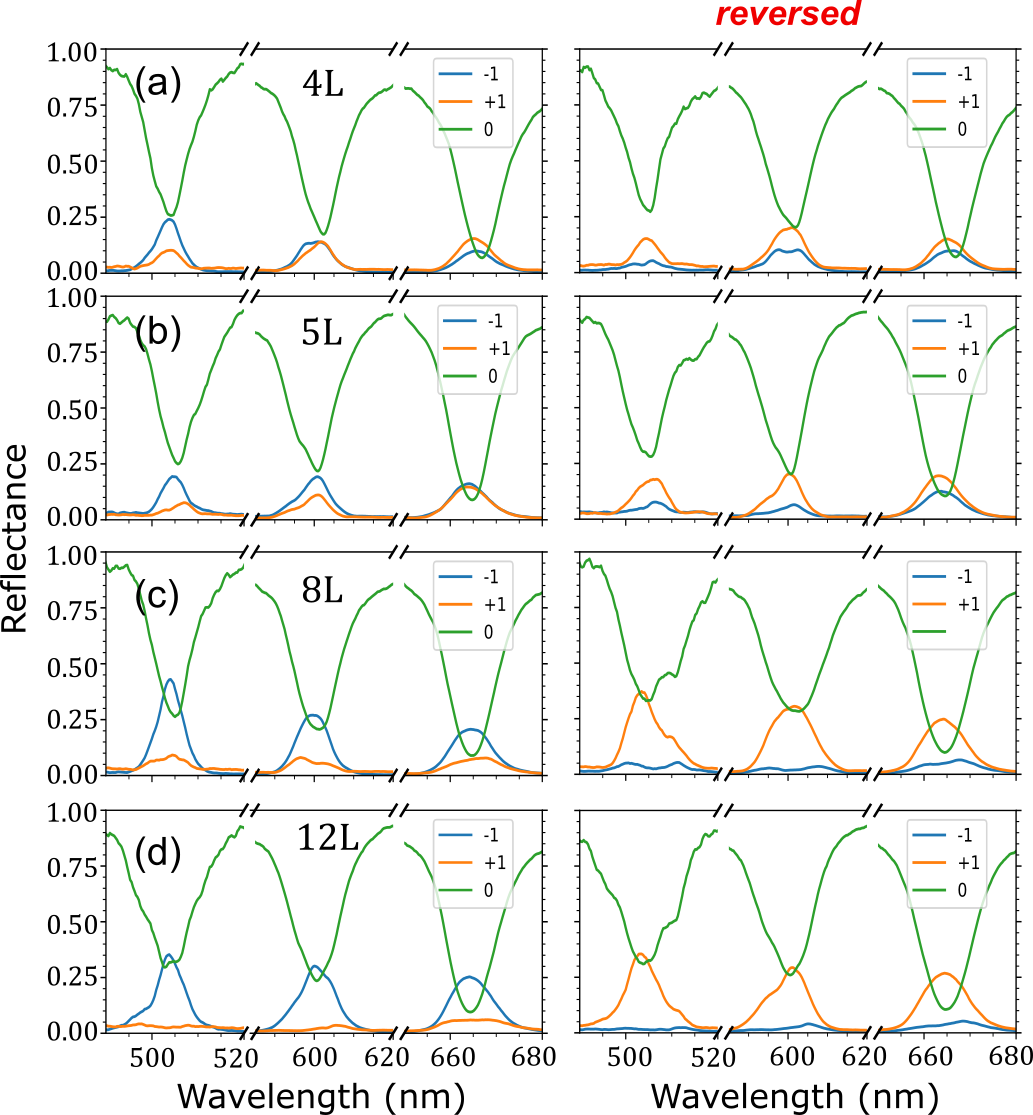


**Figure S8. Experimental efficiencies including 0^th^ order reflection:** (a) 4-pixel supercell, (b) 5-pixel supercell (5L), (c) 8-pixel supercell (8L) and (d) 12-pixel supercell (12L). The left panels correspond to the case in which the device is configured to channel power into the -1^st^ order, while the right ones correspond to the case in which the device is configured to channel power into the +1^st^ order.


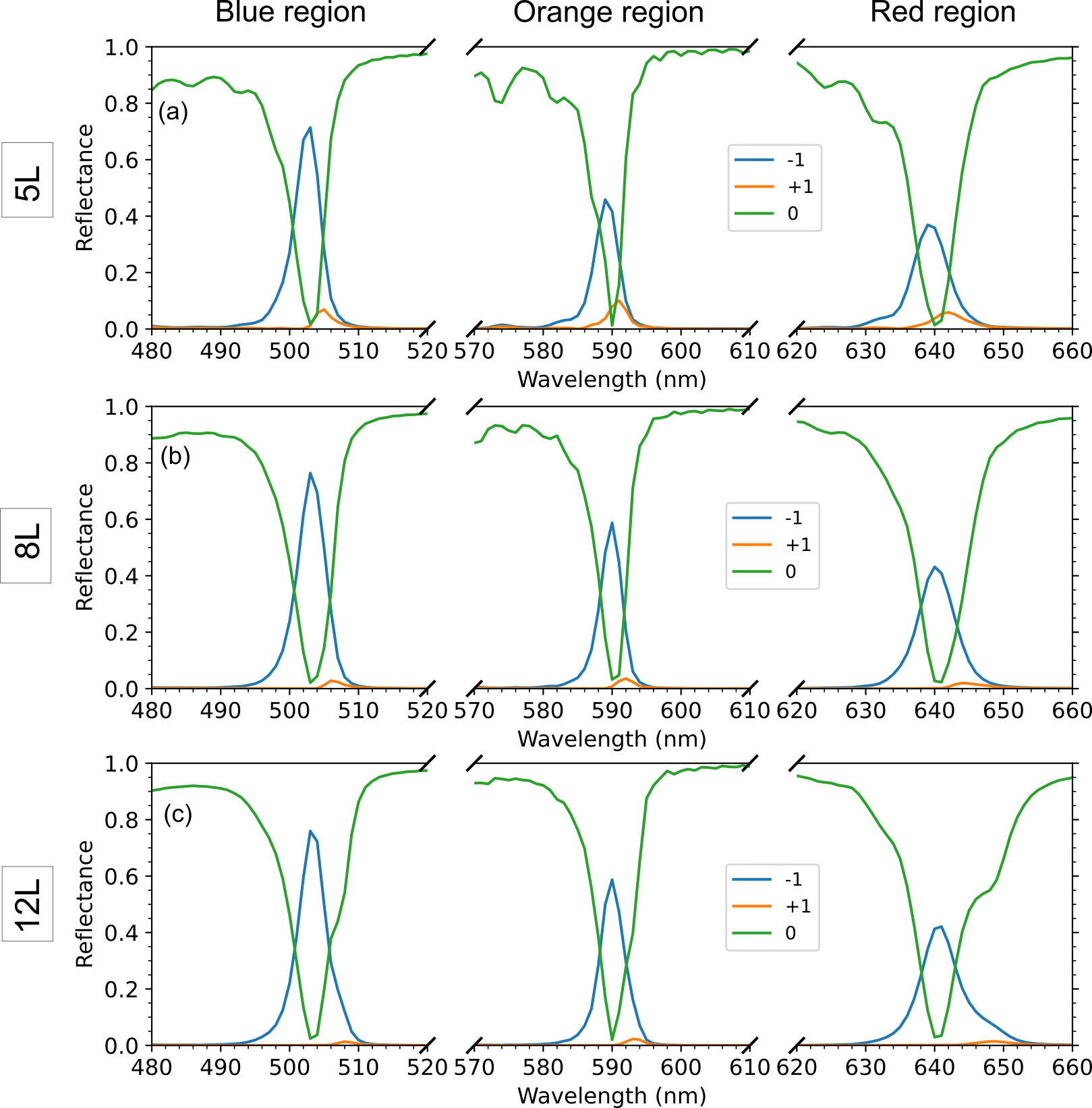


**Figure S9. Simulated efficiencies including 0^th^ order reflection:** (a) 5-pixel supercell (5L), (b) 8-pixel supercell (8L) and (c) 12-pixel supercell (12L). The linear phase (and corresponding LC angles) in simulations are optimized for 503 nm (for blue), 599nm (for orange) and 640 nm (for red).

**
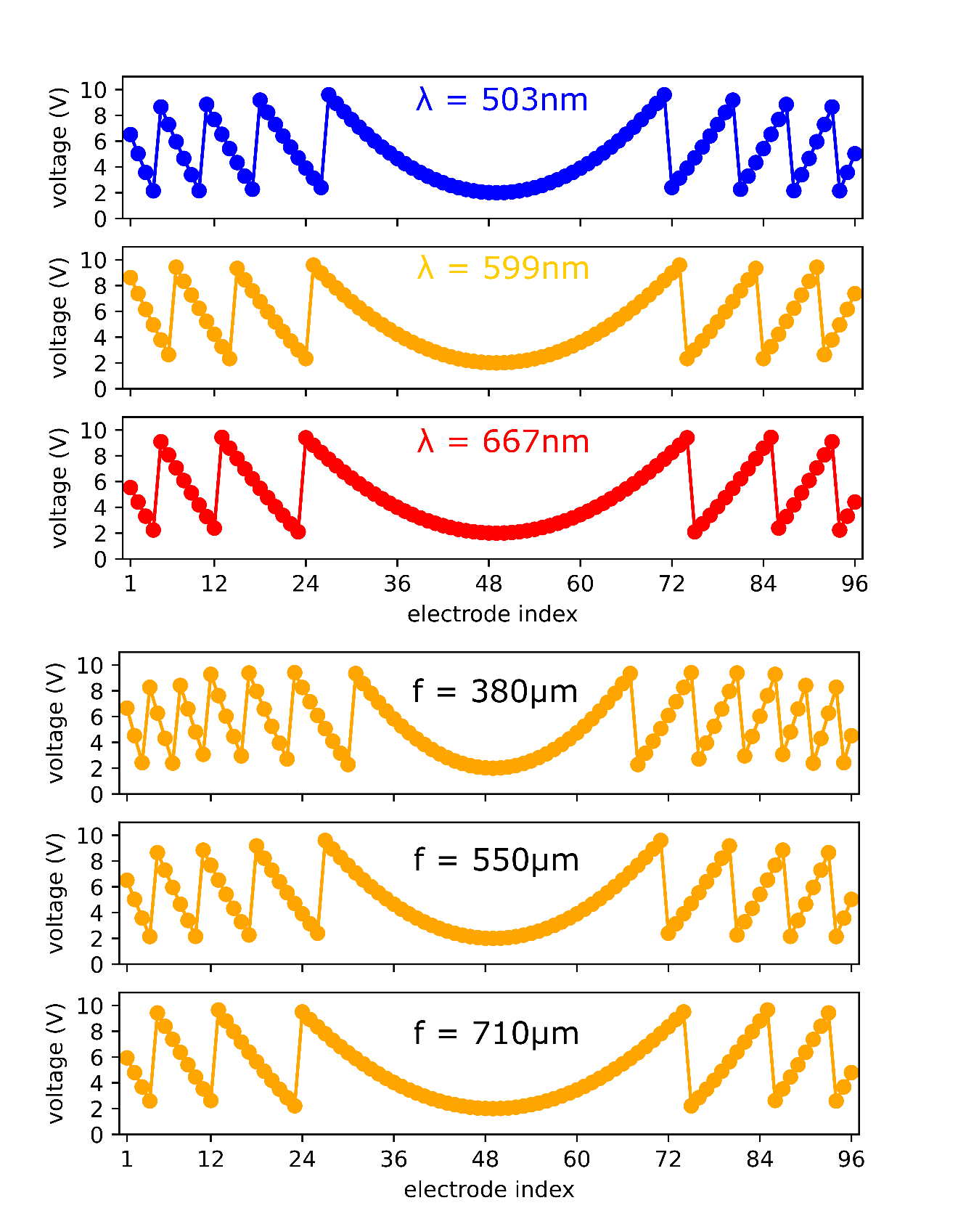
**

**Figure S10.** **Voltage profiles used for the lensing experiment.** The first three rows show voltage profiles for lensing at $z$ = 525 μm for the three target wavelengths (note that the voltage profiles were obtained for $f$= 650 μm but in experiment focusing was obtained at $z$= 525 μm). These voltage profiles are set to obtain the results presented in Fig. 5(d)-(f) in the main text. The last three rows show lensing profiles for varying focal distance corresponding to Fig. 5 (j)-(l) in the main text. Compared to the target wavelengths and focal lengths, better focusing conditions are obtained for slightly shorter wavelengths and focal distances. We attribute this difference to the fact that the voltages have not been optimized and thus the measured lensing conditions are slightly different.

**
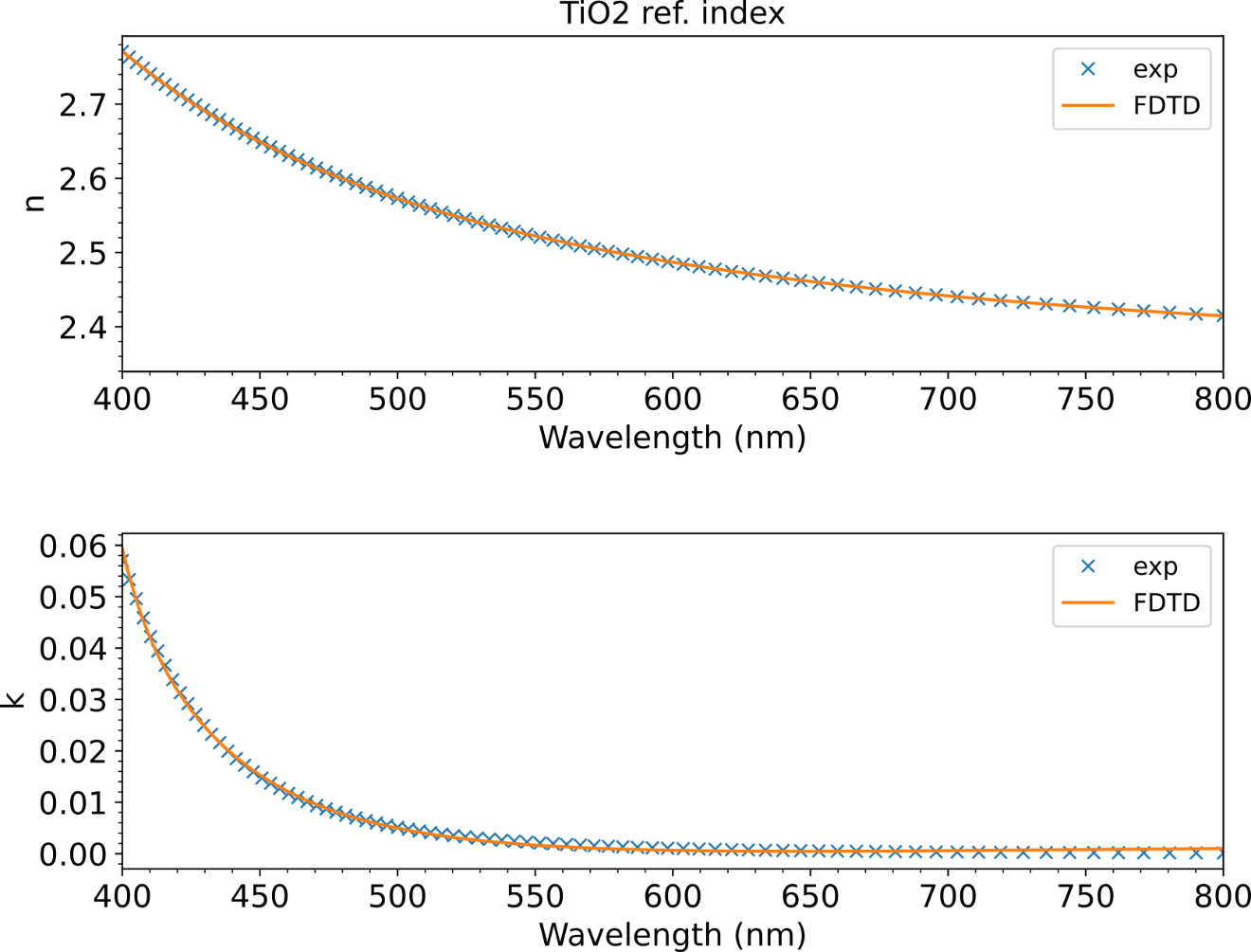
**

**Figure S11.** **Refractive index (n,k) plots for TiO_2_ used in the simulations**. Upper panel shows the real part of the refractive index (n) and the lower panel is the imaginary part (k) of refractive index. “exp” denotes the data obtained from the ellipsometry and “FDTD” denotes the fitting used in the simulations.

**
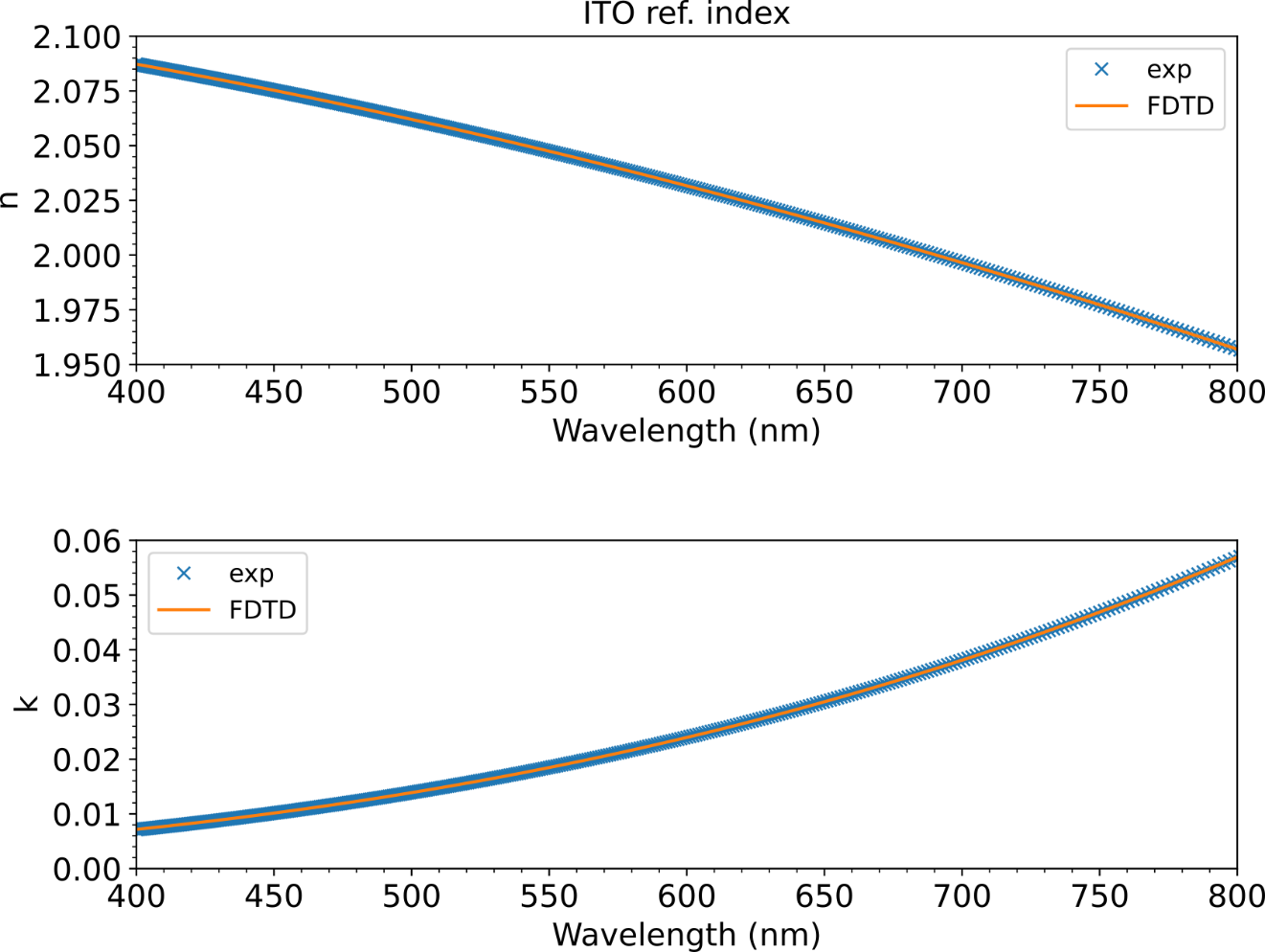
**

**Figure S12.** **Refractive index (n,k) plots for ITO (23 nm thick) used in the simulation** : Upper panel shows the real part of the refractive index (n) and the lower panel is the imaginary part (k) of refractive index. “exp” denotes the data obtained from the ellipsometry and “FDTD” denotes the fitting used in the simulations.

Captions for video files:

**Supplementary Video S1**: Switching of individual electrodes. The individual electrical switching quality of 96 electrodes in the FP-SLM sample was checked and recorded by applying a voltage of 10 Vrms at 1 kHz to each electrode sequentially under the crossed polarized excitation-collection under a microscope. The LC director was aligned parallel to the grating vector of 1D 96-electrodes and rotated to an angle of 45 degree with respect to both polarizers in the excitation and collection arms. It is shown that almost all the electrodes could be switched individually and the device could be used for beam splitting/bending testing

**Supplementary Video S2**: Spatially resolved reflectance spectra at different distances z from the device. The presented video illustrates the z-scan in Fig. 5 (k) in the main text, for which the voltage pattern with f = 650μm in Fig. S10 has been applied. It can be observed that focusing occurs only around the three operating wavelength ranges. As the voltage pattern is the same for all wavelengths in this measurement, the wavelengths are focused at slightly different z-positions, which can be observed in the video as well. With increasing distances z from the device, first the red wavelengths are being focused, then orange and finally blue.
